# *Angiostrongylus cantonensis* Is an Important Cause of Eosinophilic Meningitis in Southern Vietnam

**DOI:** 10.1093/cid/cix118

**Published:** 2017-02-04

**Authors:** Angela McBride, Tran Thi Hong Chau, Nguyen Thi Thu Hong, Nguyen Thi Hoang Mai, Nguyen To Anh, Tran Tan Thanh, Tran Thi Hue Van, Le Thi Xuan, Tran Phu Manh Sieu, Le Hong Thai, Ly Van Chuong, Dinh Xuan Sinh, Nguyen Duy Phong, Nguyen Hoan Phu, Jeremy Day, Ho Dang Trung Nghia, Tran Tinh Hien, Nguyen Van Vinh Chau, Guy Thwaites, Le Van Tan

**Affiliations:** 1 Oxford University Clinical Research Unit,; 2 University of Medicine and Pharmacy,; 3 Nguyen Trai Hospital, and; 4 Hospital for Tropical Diseases, Ho Chi Minh City, Vietnam;; 5 Centre for Tropical Medicine and Global Health, Nuffield Department of Medicine, University of Oxford, United Kingdom;; 6 Pham Ngoc Thach University of Medicine, Ho Chi Minh City, Vietnam;; 7 University College London Hospitals, United Kingdom.

**Keywords:** *Angiostrongylus cantonensis*, eosinophilic meningitis, Vietnam.

## Abstract

We utilized polymerase chain reaction (PCR) to demonstrate that *Angiostrongylus cantonensis* was responsible for 67.3% of 55 cases of eosinophilic meningitis from a cohort of 1,690 adult patients with CNS infection at a tertiary hospital in southern Vietnam. Longer duration of illness, depressed consciousness, and peripheral blood eosinophilia were associated with PCR positivity.

*Angiostrongylus cantonensis* (or “rat lung worm”) is responsible for most infectious cases of eosinophilic meningitis (EM) worldwide. *A. cantonensis* is endemic to Southeast Asia and the Pacific islands, but in recent years human cases have been reported from increasingly diverse locations [1–3]. Although the definitive host is the *Rattus* rat, humans are most frequently infected by direct ingestion of molluscan intermediate hosts (e.g., raw snails) [4]. The characteristic eosinophilic reaction occurs when parasitic larvae die in the central nervous system (CNS) of dead-end human hosts. Diagnosis is usually presumed on the basis of epidemiological risk together with cerebrospinal fluid (CSF) eosinophilic pleocytosis, but adjunctive serological assays are available in some centers. Real-time polymerase chain reaction (PCR) has recently been developed for *A. cantonensis,* but it is not yet routinely used in clinical practice, especially in resource-limited settings. Patients are usually treated with a combination of albendazole and corticosteroids, but the degree of additional benefit conferred by antihelminthic therapy is uncertain [5].

To date there have been only 8 published cases of human angiostronglyiasis from Vietnam [6]. Herein, we present the clinical features, diagnostic results by *A. cantonensis* real-time PCR, treatment, and in-hospital outcomes of 69 adults with EM in southern Vietnam.

## METHODS

Between June 2008 and January 2014, a prospective descriptive study aimed at improving the diagnosis of CNS infections was conducted in an adult infectious diseases ward at the Hospital for Tropical Diseases, a tertiary referral hospital for southern Vietnam.

Human immunodeficiency virus (HIV)-negative patients aged ≥15 years and suspected to have a CNS infection by the admitting physician were eligible for inclusion. Demographics, clinical features, laboratory results, management, and outcome were recorded. Routine CSF analysis included cell count, leucocyte differential by giemsa staining, protein, lactate and glucose analysis, gram stain and bacterial culture, india ink, and Ziehl-Neelson staining. Additional investigations included mycobacterial and fungal culture, GeneXpert for *Mycobacterium tuberculosis*, cryptococcal lateral-flow assay (IMMY, Norman, OK 73071, USA), Japanese encephalitis virus specific immunoglobulin M (IgM), real-time PCR for herpes simplex virus, and brain imaging when clinically indicated. Clinical outcomes were assessed at discharge on the basis of survival and residual symptoms relative to those on admission (recorded as: complete recovery, partial recovery, no recovery).

From the above cohort, we selected all patients with EM (defined as eosinophils ≥10% of the total CSF leucocyte count) for further analysis. We performed retrospective real-time PCR for *A. cantonensis* and conventional PCR for *Gnathostoma spinigerum* on stored CSF, with positive controls for each assay [3, 7]. We created a specificity panel from culture materials (n = 5) and CSF with other PCR-confirmed CNS pathogens (n = 26) (Supplementary Table 1). Statistical analysis was conducted using Stata (*Stata Release 9*. College Station, Texas, USA).

The study was approved by the Scientific and Ethical Committee of the Hospital for Tropical Diseases, Vietnam, and the Oxford University Tropical Research Ethics Committee, United Kingdom. Written informed consent was obtained from the patient or from a family member if the patient was unconscious.

## RESULTS

Between June 2008 and January 2014, 1,690 patients were enrolled. Results of CSF eosinophil counts by giemsa staining were available for 1,000 patients. Of 69 patients fulfilling the diagnostic criteria for EM, 55 had CSF available for PCR screening. *A. cantonensis* was detected in 37/55 (67.3%) CSF samples, whereas no sample was positive by *G. spinigerum* PCR. For *A. cantonensis* PCR positive patients, the median duration of symptoms at CSF sampling was 14 days (range 3–40 days) and the median cycle threshold (Ct) at PCR positivity was 35.99 (range 31.43–40.00). Two patients were possibly coinfected, having *A. cantonensis* and microbiological evidence of another infection (cryptococcal meningitis, *Salmonella* spp. in blood and CSF cultures). Three PCR-negative patients had alternative infections confirmed microbiologically (Supplementary Table 2). None of the 31 samples in the specificity panel yielded a detectable PCR signal.

### Demographics

Demographics, clinical features, laboratory findings, treatment, and outcome at discharge are summarized in Table 1. Male sex was predominant (n = 51, 59.4%). Twenty patients (29.0%) had a recent history of snail ingestion, and among *A. cantonensis* PCR positive patients who could identify a timeline (n = 6), the median time from last known mollusc ingestion until presentation to hospital and lumbar puncture was 20 days (range 14–56 days). There was an increase in the number of patients admitted with both eosinophilic meningitis and PCR confirmed *A. cantonensis* between June and December, the rainy season in southern Vietnam (Supplementary Figure 1).

**Table 1.** Demographic Details, Clinical Features, Results of Laboratory Investigations, Treatment Received and Outcome at Discharge for All Patients with Eosinophilic Meningitis, *A. cantonensis* PCR Positive Patients and *A. cantonensis* PCR Negative Patients

Characteristic	All eosinophilic meningitis cases (n = 69)	*A. cantonensis* PCR positive patients (n = 37)	*A. cantonensis* PCR negative patients (n = 18)	*P* value^a^
Age, years	31 (21−44)^	33 (23−44)^	22 (18−35)^	.053
Male	51 (59.4)	20 (54.1)	11 (61.1)	.624
Clinical History				
Duration of illness, days	12 (7−20)^	14 (10−20)^	9 (5−14)^	**.027**
Snail ingestion	20 (29.0)	11 (29.7)	4 (22.2)	.557
History of fever	58 (84.1)	32 (86.5)	15 (83.3)	.756
Headache ^a^	63 (96.9)	33 (97.1)	17 (100.0)	.475
Vomiting ^b^	39 (62.9)	20 (62.5)	10 (55.6)	.630
Confusion ^c^	18 (30.0)	9 (30.0)	3 (17.7)	.351
Blurred vision ^d^	5 (8.6)	5 (16.7)	0 (0.0)	.084
Speech difficulty^d^	3 (5.2)	2 (6.7)	0 (0.0)	.291
Abdominal pain^e^	5 (8.2)	5 (15.6)	0 (0.0)	.095
Examination				
GCS				.150
15	47 (68.1)	25 (67.6)	15 (83.3)	
13−14	7 (10.2)	3 (8.1)	3 (16.7)	
9−12	6 (8.7)	2 (5.4)	0 (0.0)	
6−8	9 (13.1)	7 (18.9)	0 (0.0)	**.036**
Fever ≥38°C	26 (37.7)	16 (43.2)	6 (33.3)	.481
Neck stiffness	50 (72.5)	26 (70.3)	14 (77.8)	.557
Unilatera cranial nerve palsy	6 (8.7)	4 (10.8)	2 (11.1)	
CN VI	4 (5.8)	3 (8.1)	1 (5.5)	
CN VII	2 (2.9)	1 (2.7)	1 (5.5)	
Bilateral cranial nerve palsy	3 (4.4)	2 (5.4)	1 (5.5)	
CN III & CN VI	1 (1.4)	1 (2.7)	0 (0.0)	
CN VI & CN VI	1 (1.4)	1 (2.7)	0 (0.0)	
CN VI & CN VII	1 (1.4)	0 (0.0)	1 (5.5)	
Hemiplegia^f^	2 (3.2)	1 (3.0)	1 (5.9)	
Paraplegia^f^	2 (3.2)	2 (6.1)	0 (0.0)	
Convulsions^g^	1 (1.7)	1 (3.2)	0 (0.0)	
Blood tests				
Hemoglobin g/dL	13.8 (12.9−14.8)^	13.7 (12.9−14.6)^	13.8 (13.2−15.4)^	.326
Leucocyte count ×10^9^/L	11.4 (8.9−14.6)^	10.8 (8.3−14.0)^	12.1 (9.6−15.8)^	.226
Neutrophils (%)	62.1 (51.5−75.0)^	56.6 (50.1−69.0)^	67.9 (56.4−83.9)^	**.018**
Lymphocytes (%)	14.9 (9.5−20.8)^	18.0 (11.3−23.4)^	10.2 (8.4−17.6)^	**.027**
Eosinophils (%)	13.5 (4.9−20.1)^	15.0 (7.6−23.7)^	4.2 (1.1−16.3)^	**.014**
Platelets ×10^ 9 ^ /L	307 (259−358)^	302 (260−365)^	320 (268−355)^	.905
Creatinine µmol/L	61 (49−73)^	58 (48−69)^	63 (49−74)^	.204
Serum glucose g/dL	5.4 (4.8−6.5)^	5.3 (4.8−6.2)^	5.4 (4.9−6.4)^	.930
Sodium mmol/L	132 (129−139)^	133 (129−137)^	132 (130−136)^	.896
Potassium mmol/L	3.8 (3.5−4.2)^	3.8 (3.5−4.3)^	3.8 (3.2−3.9)^	.238
CSF investigations				
Opening pressure cmH _ 2 _ 0	23 (17−33)^	22 (14−36)^	26 (18−31)^	.590
CSF appearance				.180
Clear	24 (34.8)	10 (27.0)	9 (50.0)	
Turbid	42 (60.9)	25 (67.6)	9 (50.0)	
Bloodstained	3 (4.4)	2 (5.4)	0 (0.0)	
CSF leucocyte count cells/mm^ 3 ^	564 (347−1015)^	516 (374−856)^	786 (575−978)^	.208
CSF eosinophils (%)	39 (27−52)	39 (28−48)	42 (30−58)	.394
CSF protein g/dL	0.8 (0.5−1.2)^	0.9 (0.6−1.1)^	0.7 (0.5−1.2)^	.693
CSF protein>0.5 g/dL	55 (80.8)	30 (81.1)	16 (88.9)	.463
CSF glucose g/dL	2.5 (1.9−2.8)^	2.4 (2.0−2.7)^	2.6 (1.7−2.9)^	.533
CSF/blood glucose <0.5	38 (55.0)	21 (56.8)	9 (50.0)	.637
CSF lactate mmol/L	2.8 (2.2−3.9)^	2.9 (2.5−3.7)^	2.6 (2.0−4.0)^	.264
Treatment^h^				
Albendazole	50 (83.3)	29 (93.5)	11 (68.8)	**.024**
Corticosteroids	50 (83.3)	26 (83.9)	13 (81.3)	.821
Ceftriaxone	17 (28.3)	7 (22.6)	5 (31.3)	.518
Other antimicrobial	10 (16.7)	5 (16.1)	4 (25.0)	.464
Outcome at discharge^i^				.532
Full recovery	18 (29.0)	9 (28.1)	8 (50.0)	
Partial recovery	39 (62.9)	20 (62.5)	8 (50.0)	
No recovery	4 (6.5)	2 (6.3)	0 (0.0)	
Death	1 (1.6)	1 (3.1)	0 (0.0)	

n (%), median (25th centile−75th centile).

Abbreviations: CSF, cerebrospinal fluid; PCR, polymerase chain reaction.

^a^ *P* values are presented for comparison of parameters between PCR positive and negative patients: statistical tests included Pearsons *χ*^2^, unpaired *T*-test and Wilcoxon-Mann–Whitney tests. *P* values less than 0.05 are highlighted in bold type. Where data were not available for all patients, superscript indicates n for all eosinophilic meninigitis patients, *A. cantonensis* PCR positive, and *A. cantonensis* PCR negative patients respectively: a n = 65,34,17, b n = 62,32,18, c n = 60,30,17, d n = 58,30,16, e n = 61,30,16, f n = 62,33,17, g n = 60,31,16, h n = 60,31,16, i n = 62,32,16. GCS (Glasgow coma scale). CN (cranial nerve).

### Clinical Features

Most patients presented with headache (n = 63, 96.9%), fever (n = 58, 84.1%), and vomiting (n = 39, 62.9%). At presentation, 50 (72.5%) patients had neck stiffness, 20 (37.7%) had fever ≥38°C, and 9 (12.1%) patients had cranial nerve palsy: 6 unilateral and 3 bilateral. The most common nerve affected was CN VI. Nine patients were deeply comatose (GCS ≤ 8) on admission.

*A. cantonensis* PCR positive patients had a longer duration of illness at presentation (median 14 days, interquartile range (IQR) 10–20 vs. 9 days, IQR 5–14, *P* = .027), and were more likely to present comatose (7 (18.9%) vs. 0(0.0%), *P* = .036) than PCR negative patients. Severity of presentation was not related to Ct at PCR positivity (Supplementary Table 3). PCR positive patients had higher peripheral blood eosinophil (median 15.0%, IQR 7.6–23.7 vs. 4.2%, 1.1–16.3, *P* = .014) and lymphocyte percentages (median 18.0%, IQR 11.3–23.4 vs. 10.2%, 8.4–17.6, *P* = .027), and lower neutrophil percentages (median 56.6%, IQR 50.1–69 vs. 67.9%, 56.4–83.9, *P* = .018) than PCR negative patients. Results of CSF microscopy and biochemical analysis did not differ significantly between the 2 patient groups.

In sum, 11 patients with *A. cantonensis* PCR positive CSF underwent CNS imaging by computed tomography (CT) +/− magnetic resonance imaging (MRI). Abnormalities included: cerebral edema (5/11), meningeal inflammation (4/11), and focal parenchymal hyper-intense lesions on T2-weighted FLAIR MRI (5/11).

### Treatment and Outcome

Fifty patients (86.7%) were treated with dexamethasone and albendazole. One patient died during admission; he was 78 years old and had concurrent disseminated salmonellosis. Of the surviving patients, 43 (69.4%) had residual symptoms at discharge, but the severity or longevity of residual symptoms was not evaluated. Univariate analysis indicated that risk factors for adverse outcome (defined as no recovery or death) included age in the upper quartile (44–78 years) (Odds Ratio (OR) 13.5, 95% CI 1.4–131.9, *P* = .025) and GCS ≤ 8 at presentation (OR 19.9, 95% CI 2.5–155.6, *P* = .004). Due to the rarity of adverse outcome, we did not proceed to multivariate logistic regression. There was no significant difference in outcome between *A. cantonensis* PCR positive and negative patients.

## DISCUSSION

Here we have described the presenting features, laboratory results, and outcomes for patients with EM in southern Vietnam. To date, there have been only 8 cases of angiostrongyliasis published from Vietnam [4, 8–10]. We have applied real-time PCR to confirm that *A. cantonensis* is an important cause of EM in Vietnam, accounting for 67.3% of cases in our series; meanwhile we found no evidence for *G. spinigerum* as a cause of EM.

Consistent with the clinical features described in previous reports, our patients presented with an extended prodrome of headache, fever, and vomiting [6]. Although PCR positivity was not related to the results of routine CSF microscopy or biochemistry, we found that patients who tested positive for *A. cantonensis* by PCR typically had a higher peripheral blood eosinophil percentage, lower neutrophil percentage, a longer duration of illness, and were more likely to present in deep coma than those who tested negative.

EM in adults is usually mild and self-limiting [6]. Likewise, we observed that only one patient died during admission. A case series of Thai patients admitted with eosinophilic meningoencephalitis found that 10/11 patients who presented in coma died [11]. However, in our setting, although comatose patients were significantly more likely to have an adverse outcome, 4/9 achieved either full or partial recovery.

Most of our patients were treated with a combination of albendazole and corticosteroids, but there is no definitive evidence for the use of anti-helminthic agents. Chotmongkol et al found no additional benefit of 14 days albendazole plus prednisolone as compared to prednisolone alone in reducing the duration of headache in EM; however, this study did not reach the planned sample size [5]. Adequately powered randomized controlled trials are needed to guide the optimal management of EM.

Our study has some limitations. First, children aged <15 years were not included. Second, most patients are only referred to our hospital following a failure to improve at peripheral hospitals. Third, only 1,000/1,690 patients enrolled with CNS infections had CSF eosinophil counts available for assessment, and patient selection based only on eosinophil counts of acute CSF samples may have been suboptimal [12]. Fourth, although an optimal diagnostic algorithm for *A. cantonensis*-associated EM has not been established, PCR/serological testing of serial CSF samples may have improved the diagnostic yield [3, 12]. Fifth, patients were not followed up beyond discharge. Hence, our data may underrepresent the true burden of *A. cantonensis* meningitis and/or overestimate the severity of the clinical spectrum.

## CONCLUSIONS

For the first time we have shown by specific PCR that *A. cantonensis* is a major cause of EM in adults in southern Vietnam. Further studies including children are needed to evaluate the true burden of *A. cantonensis* meningitis in Vietnam, and assess the optimal diagnostic and treatment strategies for this disease.

## Supplementary Data

Supplementary materials are available at *Clinical Infectious Diseases* online. Consisting of data provided by the authors to benefit the reader, the posted materials are not copyedited and are the sole responsibility of the authors, so questions or comments should be addressed to the corresponding author.

## Supplementary Material

Supplementary_Table_1_2_3_and_Figure_1

## Notes

***Acknowledgements.*** We thank the staff of Viet-Anh ward, who helped care for the patients and collect clinical data, in particular nurses Pham Thi Diep, Pham Thi Kim, Nguyen Hoa Trang, Nguyen Thi Ly, and Ms Le Kim Thanh.

***Financial support.*** This work was supported by the Wellcome Trust (101104/Z/13/Z, 089276/Z/09/Z, WT097147MA).

***Potential conflicts of interest.*** All authors: No reported conflicts. All authors have submitted the ICMJE Form for Disclosure of Potential Conflicts of Interest. Conflicts that the editors consider relevant to the content of the manuscript have been disclosed.
