# Supplementary material for: Angiostrongylus cantonensis Is an Important Cause of Eosinophilic Meningitis in Southern Vietnam
Source: Clin Infect Dis. 2017 Feb 4;64(12):1784–7. doi: 10.1093/cid/cix118 (PMC5447893; doi:10.1093/cid/cix118)
Supplement: Supplementary_Table_1_2_3_and_Figure_1 [file cix118_suppl_Supplementary_Table_1_2_3_and_Figure_1.docx]

Supplementary Table 1. Components of specificity panel constructed from CSF of patients with PCR confirmed CNS infections, and extracted DNA from culture materials.

| Organism | Number of samples |
| --- | --- |
| *Streptococcus suis* | 19 |
| *Streptococcus pneumoniae* | 3 |
| Herpes simplex virus | 2 |
| *Haemophilus influenzae* | 1 |
| Dengue virus | 1 |
| *Cryptococcus gattii** | 1 |
| *Cryptococcus neoformans** | 1 |
| *Mycobacterium tuberculosis** | 1 |
| *Salmonella typhimurium** | 1 |
| *Salmonella typhi** | 1 |

*indicates DNA was extracted from culture materials rather than CSF

Supplementary Table 2. Laboratory results and outcomes for patients with microbiologically confirmed non-parasitic infection or co-infection.

| Alternative diagnosis | Supporting microbiology | *A. cantonensis* PCR result (Cycle threshold) | CSF eosinophil count (% total white cells) | Outcome |
| --- | --- | --- | --- | --- |
| *Salmonella* spp meningitis and bacteraemia | CSF and blood culture positive for *Salmonella* spp. | Positive (Ct 34) | 75% | Died |
| Cryptococcal meningitis | CSF india ink and Cryptococcal lateral flow assay positive | Positive (Ct 35) | 42% | Partial recovery |
| *Streptococcus suis* meningitis | CSF gram stain and culture positive | Negative | 24% | Partial recovery |
| Tuberculous meningitis | CSF Ziehl-Neelson smear positive | Negative | 30% | Partial recovery |
| Tuberculous meningitis | CSF Ziehl-Neelson smear positive | Negative | 18% | Referred to TB specialist hospital |

Supplementary Table 3. Comparison of markers of severity and occurrence of adverse outcome for patients with lower Ct values (≤35.99) and higher Ct values (>35.99) for *A. cantonensis* PCR. P values are presented for comparison of proportions by Chi-squared analysis.

| Marker of severity | *A. cantonensis* PCR Ct≤35.99 (n=19) | *A. cantonensis* PCR Ct>35.99 (n=18) | P value |
| --- | --- | --- | --- |
| GCS<15 at presentation | 5 (26.3%) | 7 (38.9%) | 0.41 |
| GCS≤8 at presentation | 3 (15.8%) | 4 (22.2%) | 0.62 |
| Fever≥38⁰C at presentation | 7 (36.8%) | 9 (50.0%) | 0.42 |
| Adverse outcome (no recovery/death) | 1 (5.2%) | 2 (11.1%) | 0.47 |

Supplementary Figure 1. Scatter graph indicating the number of patients admitted by month, pooled across all years of the study for all patients with eosinophilic meningitis (diamond), and *A. cantonensis* PCR positive patients (square).
